# Oxidative Stress in Xenograft Mouse Model Exposed to Dendrimers Decorated Polydopamine Nanoparticles and Targeted Chemo- and Photothermal Therapy

**DOI:** 10.3390/ijms242316565

**Published:** 2023-11-21

**Authors:** Marta Witkowska, Radosław Mrówczyński, Bartosz Grześkowiak, Izabela Miechowicz, Ewa Florek

**Affiliations:** 1 Laboratory of Environmental Research, Department of Toxicology, Poznan University of Medical Sciences, Rokietnicka 3, 60-806 Poznań, Poland; witkowskamr@gmail.com; 2 Faculty of Chemistry, Adam Mickiewicz University, Uniwersytetu Poznańskiego 8, 61-614 Poznań, Poland; 3 Centre for Advanced Technologies, Adam Mickiewicz University, Uniwersytetu Poznańskiego 10, 61-614 Poznań, Poland; 4 NanoBioMedical Centre, Adam Mickiewicz University in Poznan, Uniwersytetu Poznańskiego 3, 61-614 Poznań, Poland; bartosz.grzeskowiak@amu.edu.pl; 5 Department of Computer Science and Statistics, Poznan University of Medical Sciences, 60-806 Poznań, Poland; iza@ump.edu.pl

**Keywords:** polydopamine, nanoparticles, in vivo, oxidative stress, photothermal therapy, targeted therapy, chemotherapy

## Abstract

Polydopamine (PDA)-based nanostructures are used for biomedical purposes. A hybrid drug nanocarrier based on a PDA decorated with polyamidoamine (PAMAM) dendrimers G 3.0 (DG3) followed by a connection with glycol (PEG) moieties, folic acid (FA), and drug doxorubicin (DOX) was used for combined chemo- and photothermal therapy (CT-PTT) of liver cancer. Oxidative stress plays a crucial role in the development of cancer, and PDA seems to have the ability to both donate and accept electrons. We investigated oxidative stress in organs by evaluating oxidative stress markers in vivo. In the liver, the level of reduced glutathione (GSH) was lower and the level of Trolox equivalent antioxidant capacity (TEAC) was higher in the group receiving doxorubicin encapsulated in PDA nanoparticles with phototherapy (PDA@DG3@PEG@FA@DOX + PTT) compared to the control group. The concentration of thiobarbituric acid reactive substances (TBARS) in livers, was higher in the group receiving PDA coated with PAMAM dendrimers and functionalized with PEG and FA (PDA@DG3@PEG@FA) than in other groups. Markers in the brain also showed lower levels of GSH in the PDA@DG3@PEG@FA group than in the control group. Markers of oxidative stress indicated changes in the organs of animals receiving PDA nanoparticles with PAMAM dendrimers functionalized with FA in CT-PTT of liver cancer under in vivo conditions. Our work will provide insights into oxidative stress, which can be an indicator of the toxic potential of PDA nanoparticles and provide new strategies to improve existing therapies.

## 1. Introduction

The wide application of polydopamine (PDA)-based structures in the preparation of multifunctional nanoparticles for biomedical purposes is due to its unique properties such as cheap and easy preparation via oxidative polymerization in alkali solution, proven biocompatibility, and strong adhesive properties that allow its deposition on different nanomaterials both in macro and nanoscale [1,2,3,4].

Moreover, a wide spectrum of versatile reactions for PDA surface functionalization, e.g., reactions with amines and thiols [5,6] or by click chemistry [7] and ring-opening polymerization [8], low-cost preparation methods based on oxidative polymerization in basic solutions, the possibility of size tuning and achieving different PDA shapes, stability in biological media, and chelating properties allowing binding to almost all transition metals and radioisotopes. However, one of the most important features of PDA for medical applications is its relatively high photothermal conversion efficiency (40%) in the I biological window at 808 nm [9]. This attribute makes PDA and PDA-coated materials valuable photothermal agents. Therefore, PDA nanostructures have been used as versatile nanomaterials capable of merging different modalities for oncological purposes. Especially, merging chemo- and photothermal therapy (CT-PTT), photothermal therapy with photodynamic (PTT-PD), and gene therapy with PTT have drawn the attention of many scientific groups [10,11]. In the research conducted by Jędrzak as well as Mrówczyński, it has been demonstrated that the PDA-coated magnetic nanoparticles functionalized with mono-6-thio-β-cyclodextrin or polyamidoamine (PAMAM) dendrimers and their application in combined chemo- and photothermal therapy towards liver cancer cells in vitro [12,13]. Lately, they reported that the synthesis of a hybrid drug nanocarrier based on PDA nanoparticles decorated with PAMAM dendrimers was also suitable for combined CT-PTT of liver cancer cells in vitro. Moreover, in this report, a universal strategy for peripheral amino group functionalization using a bifunctional linker via thiol-ene click chemistry to ensure the active targeting of cancer cells was presented [4].

What is important is that oxidative stress plays a crucial role in the development of cancer, including liver cancer [14]. In rapidly dividing and developing cells, its level is very high, which makes the pathological creation have to deal with its intensity. However, even in this case, when the level of oxidative stress increases even more, the cells are not able to neutralize it [15]. For this reason, it is beneficial to use PDA nanomaterials because their properties allow them to be used in treatment where it is necessary to reduce the level of reactive oxygen species (ROS) [16].

PDAs have the ability to both donate and accept electrons. This property depends on the redox state in which they occur such as reduced catechol, intermediate semiquinone radical, and oxidized o-quinone [17]. This contributes to different effects on free radicals. The ability to donate electrons favors the formation of ROS, while accepting them by the nanomaterial neutralizes the ROS. These properties happen due to the presence of catechol groups contained in PDA and their ability to occur in various degrees of oxidation. The state in which PDA occurs also depends on the influence of external factors, e.g., binding to metals or exposure to radiation, which is used in phototherapy [18]. It has been shown by Liu et al. that PDA can behave as a radical scavenger to O_2_^•−^. The process is similar to that of superoxide dismutase catalyzing O_2_^•−^ to O_2_ by observing the appearance of a large number of bubbles and subsequently monitoring the kinetics of O_2_ generation to obtain a typical Michaelis–Menten kinetic curve; however, the scavenging effect of PDA on O_2_^•−^ is not affected by the change in pH [19]. It has been proposed that the reaction occurs as follows:

DA^•^ + O_2_^•−^ → PDA^−^ + O_2_

PDA^−^ + O_2_^•−^ + 2H_2_O → PDA^•^ + H_2_O_2_ + 2OH^−^

It is also believed that PDA may block a Fenton reaction by chelating metal ions like Cu^+^ or Fe^2+^. Therefore, the PDA has already found application as a radical scavenger in biological applications. The electron taken from oxygen by PDA causes it to transform into a radical, which results in the formation of other ROS in further reactions, e.g., H_2_O_2_. Analogous to the oxidation process, an electron is released during the reduction of the above-mentioned states of the catechol moiety. It can be transferred to ROS, causing them to become stable and lose their ability to react with proteins or lipids [18,20].

Here, we present the studies on the generation of ROS in kidneys, livers, and brains by evaluating oxidative stress markers including total protein (TP), reduced glutathione (GSH), glutathione S-transferase (GST), thiobarbituric acid reactive substances (TBARS), Trolox equivalent antioxidant capacity (TEAC), nitric oxide (NO), and catalase (CAT). Thus, the aim was to evaluate the toxicity of combined chemo- and photothermal therapy employing PDA decorated with polyamidoamine (PAMAM) dendrimers and chemotherapeutic drugs by assessing oxidative stress in this organ of xenograft mice using well-known oxidative stress biomarkers. An understanding of oxidative stress from these nanoparticle structures combined with CT-PTT can improve existing therapies to treat liver cancer, create new strategies to improve therapeutic outcomes, and allow an understanding of the influence of PDA-based therapy on animals. Thus, the presented data are important in terms of the translation potential of PDA materials in future anticancer therapies.

## 2. Results

**Figure 1:**
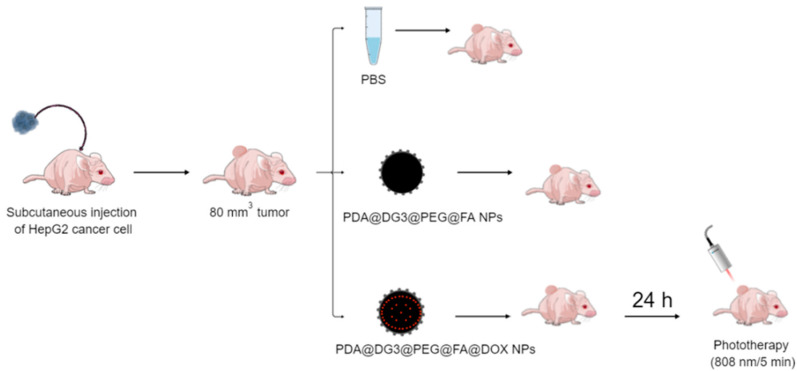
Schematic representation of the designed experiment.

The experimental approach is illustrated in Figure 1. Briefly, the polydopamine nanoparticles modified with PAMAM dendrimers are from now on described as PDA@DG3@PEG@FA, or after doxorubicin loading PDA@DG3@PEG@FA@DOXO; they were administrated intravenously to immunodeficient mice. After exposure to 808 nm NIR light, we evaluated the series of important stress markers such as body weight, which are important toxicological parameters. The data are presented below.

### 2.1. Body Weights and Clinical Signs

**Figure 2:**
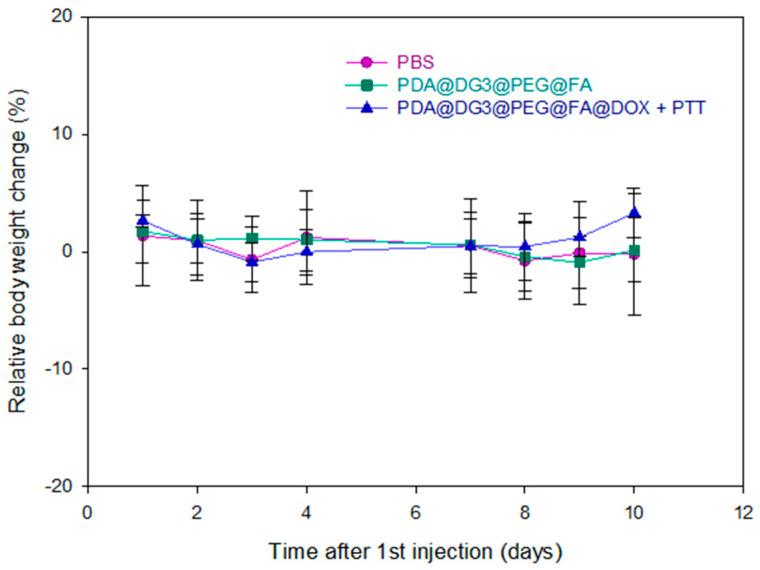
Relative body weight change of mice as a function of time after IV injection of PBS, PDA@DG3@PEG@FA, and PDA@DG3@PEG@FA@DOX + PTT. The values are expressed as means ± SD.

Measuring body weight is an important parameter to help us obtain early information on the toxicity of test materials. Figure 2 shows the body weight variations of the 18 female BALB/c nude mice after receiving different treatment methods. It can be seen that the treatment of intravenously injected therapy with PDA@DG3@PEG@FA and PDA@DG3@PEG@FA@DOX + PTT caused no mortality and no relative body weight change as mice lost no more than 10% of their body weight throughout the efficacy study. Clinical signs and symptoms were also not observed in the treated mice (Figure 2). 

### 2.2. Concentrations of Oxidative Stress Markers and Biochemical Parameters

#### 2.2.1. Determination of Total Protein (TP) and Nitric Oxide (NO) Concentration

**Figure 3:**
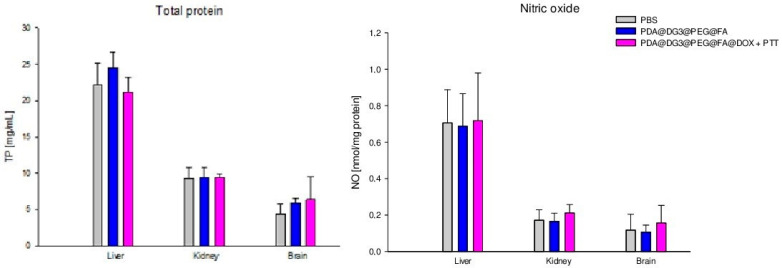
Comparison of total protein (TP) and nitric oxide (NO) concentrations in the liver, kidneys, and brain of animals exposed to PBS, PDA@DG3@PEG@FA, and PDA@DG3@PEG@FA@DOX + PTT.

The highest concentration of total protein (TP) and nitric oxide NO was found in the liver. However, the administration of nanoparticles and further therapy did not cause an increase in both markers in the liver, brain, or kidneys. Moreover, total protein and nitric oxide levels between all groups showed no statistically significant differences (Figure 3).

#### 2.2.2. Determination of Reduced Glutathione (GSH) Concentration

**Figure 4:**
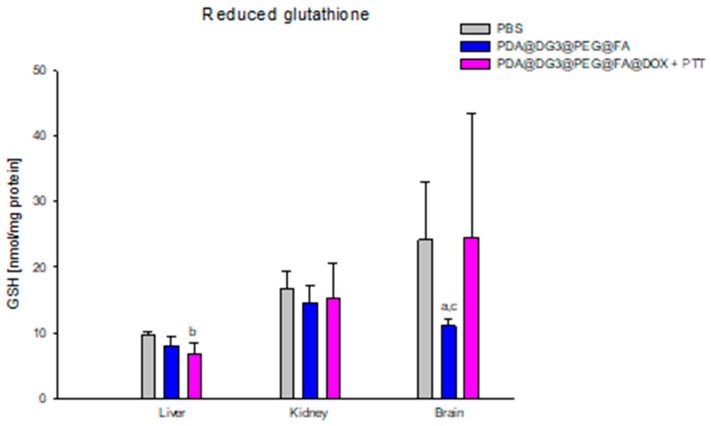
Comparison of reduced glutathione (GSH) concentrations in the liver, kidneys, and brain of animals exposed to PBS, PDA@DG3@PEG@FA, and PDA@DG3@PEG@FA@DOX + PTT. (a) Statistically significant difference between the group of animals receiving polydopamine nanoparticles and the animals from the control group (*p* = 0.000556); (b) statistical significance between the group of animals receiving polydopamine nanoparticles with the drug and the use of phototherapy and the animals from the control group (*p* = 0.000029); (c) statistically significant difference between the group of animals receiving polydopamine nanoparticles with the drug and the use of phototherapy and the animals receiving polydopamine nanoparticles (*p* = 0.027011).

GSH concentration in the liver of mice from the PDA@DG3@PEG@FA@DOX + PTT group (6.72 ± 1.71 nM/mg protein) was significantly lower than the values obtained in the control group, PBS (9.69 ± 0.52 nM/mg protein), by 30.7% (Figure 4). In the tested animal brain, statistical analysis showed a significant difference in the concentration of GSH in animals from the PDA@DG3@PEG@FA group (11.03 ± 1.11 nM/mg protein) compared to animals from the PBS group (24.23 ± 8.72 nM/mg protein), which was lower by 45.54%. There was also a significant difference between the values obtained in the brain of mice from the PDA@DG3@PEG@FA group (11.03 ± 1.11 nM/mg protein) and the PDA@DG3@PEG@FA@DOX + PTT group (26.50 ± 12.66 nM/mg protein), where the concentration of GSH was 40.17% higher in the brain of animals from the PDA@DG3@PEG@FA group (Figure 4).

#### 2.2.3. Determination of Lipid Peroxidation (TBARS) Concentration

**Figure 5:**
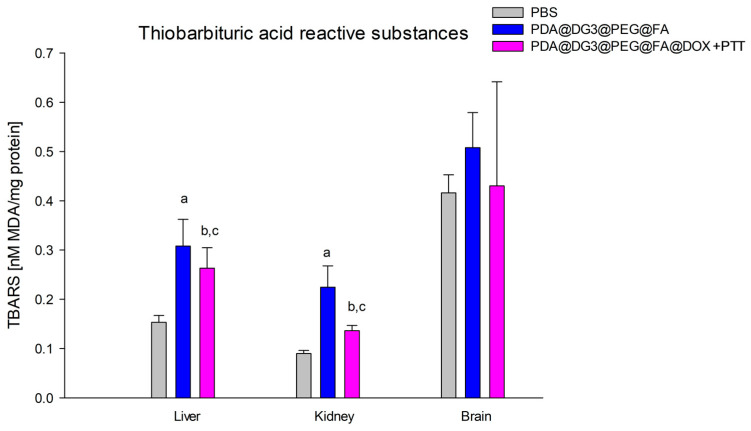
Comparison of thiobarbituric acid reactive substances (TBARS) concentrations in the liver, kidneys, and brain of animals exposed to PBS, PDA@DG3@PEG@FA, and PDA@DG3@PEG@FA@DOX + PTT. (a) Statistically significant difference between the group of animals receiving polydopamine nanoparticles and the animals from the control group (liver *p* =< 0.000001; kidney *p* =< 0.000001); (b) statistical significance between the group of animals receiving polydopamine nanoparticles with the drug and the use of phototherapy and the animals from the control group (liver *p* = 0.000029; kidney *p* = 0.000452); (c) statistically significant difference between the group of animals receiving polydopamine nanoparticles with the drug and the use of phototherapy and the animals receiving polydopamine nanoparticles (liver *p* = 0.048163; kidney *p* = 0.002551).

Our studies of liver concentration showed statistically significant differences in lipid peroxidation of mice from the PDA@DG3@PEG@FA group (0.31 ± 0.05 nM MDA/mg protein) in relation to two groups: control, PBS (0.15 ± 0.01 nM MDA/mg protein), and the PDA@DG3@PEG@FA@DOX + PTT group (0.26 ± 0.04 nM MDA/mg protein). Lipid peroxidation was significantly higher by 51.6% and 16.1%, respectively, in contrast to the compared groups. TBARS concentration was significantly higher in the PDA@DG3@PEG@FA@DOX + PTT group (0.26 ± 0.04 nM MDA/mg protein) compared to the values obtained in the liver of PBS mice (0.15 ± 0.01 nM MDA/mg protein) by 42.3% (Figure 5). This study also showed statistically significant differences in lipid peroxidation activity in the kidneys of mice from the group PDA@DG3@PEG@FA (0.22 ± 0.04 nM MDA/mg protein) compared to the PBS group (0.09 ± 0.01 nM MDA/mg protein). TBARS activity after intravenous administration of nanomaterials was significantly higher by 59.09% compared to animals from the control group. TBARS activity was significantly higher in animals from the PDA@DG3@PEG@FA@DOX + PTT group (0.14 ± 0.01 nM MDA/mg protein) compared to the PBS group (0.09 ± 0.01 nM MDA/mg protein), where TBARS activity in kidneys of animals from the PDA@DG3@PEG@FA@DOX + PTT group was higher by 35.71%. There was also a statistically significant decrease in TBARS activity in the kidneys of mice from the PDA@DG3@PEG@FA@DOX + PTT group (0.14 ± 0.01 nM MDA/mg protein) by 36.37% compared to the PDA@DG3@PEG@FA group (0.22 ± 0.04 nM MDA/mg protein) (Figure 5).

#### 2.2.4. Determination of Antioxidant Capacity Trolox Equivalents (TEAC)

**Figure 6:**
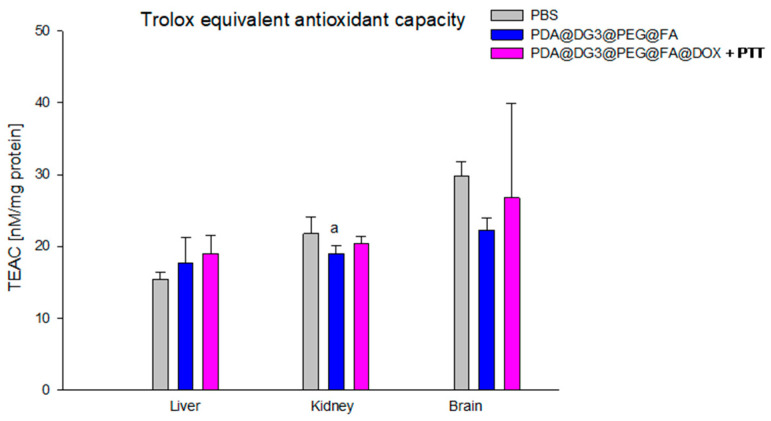
Comparison of antioxidant capacity of Trolox equivalents (TEAC) concentrations in the liver, kidneys, and brain of animals exposed to PBS, PDA@DG3@PEG@FA, and PDA@DG3@PEG@FA@DOX + PTT. (a) Statistically significant difference between the group of animals receiving polydopamine nanoparticles and the animals from the control group (*p* = 0.008034).

Our study showed a borderline statistically significant result in the total antioxidant capacity of Trolox equivalents in the liver. The TEAC of PDA@DG3@PEG@FA@DOX + PTT mice (18.98 ± 2.58 nM/mg protein) versus the PBS group (15.43 ± 0.95 nM/mg protein) was 18.7% higher. Statistically significant differences were also seen in TEAC level in the kidneys of PDA@DG3@PEG@FA mice (19.01 ± 1.02 nM/mg protein) versus PBS animals (21.80 ± 2.29 nM/mg protein), and the results showed that the total antioxidant capacity of Trolox equivalents in the kidneys of mice receiving PDA@DG3@PEG@FA was lower by 12.80% (Figure 6).

#### 2.2.5. Determination of S-Transferase Activity (GST) Concentration

**Figure 7:**
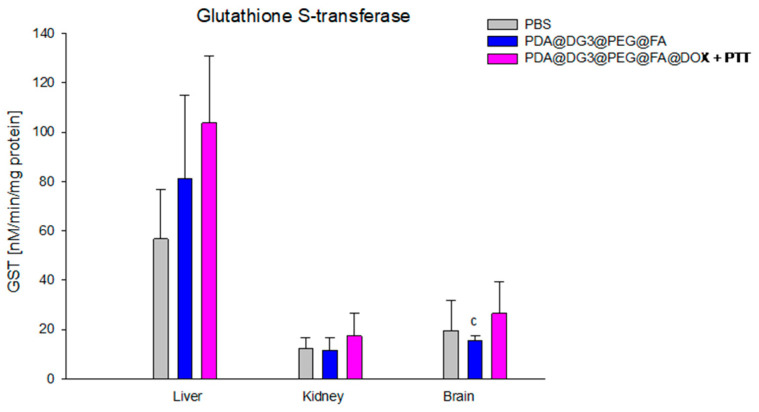
Comparison of S-transferase activity (GST) concentrations in the liver, kidneys, and brain of animals exposed to PBS, PDA@DG3@PEG@FA, and PDA@DG3@PEG@FA@DOX + PTT. (c) Statistically significant difference between the group of animals receiving polydopamine nanoparticles with the drug and the use of phototherapy and the animals receiving polydopamine nanoparticles (*p* = 0.043361).

The average activity of glutathione S-transferase in the brain of mice from the PDA@DG3@PEG@FA@DOX + PTT group (26.50 ± 12.66 nM/min/mg protein) was significantly higher, by as much as 72.25%, compared to animals from the PDA@DG3@PEG@FA group (15.39 ± 1.91 nM/min/mg protein) (Figure 7).

#### 2.2.6. Determination of Catalase (CAT) Concentration

**Figure 8:**
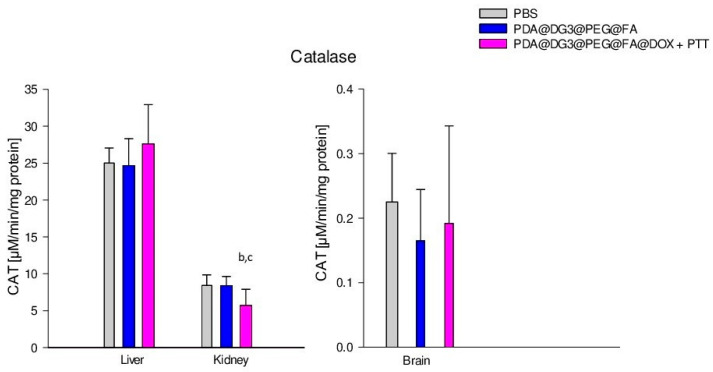
Comparison of catalase (CAT) concentrations in the liver, kidneys, and brain of animals exposed to PBS, PDA@DG3@PEG@FA, and PDA@DG3@PEG@FA@DOX + PTT. (b) Statistical significance between the group of animals receiving polydopamine nanoparticles with the drug and the use of phototherapy and the animals from the control group (*p* = 0.00842); (c) Statistically significant difference between the group of animals receiving polydopamine nanoparticles with the drug and the use of phototherapy and animals receiving polydopamine nanoparticles (*p* = 0.007692).

Our study showed statistically significant differences in catalase activity in the kidneys of mice from the PDA@DG3@PEG@FA@DOX + PTT group (5.69 ± 2.20 µM/min/mg protein) compared to the PBS group (8.43 ± 1.42 µM/min/mg protein). CAT activity in the PDA@DG3@PEG@FA@DOX + PTT group was significantly lower by 32.50% compared to animals in the PBS group. CAT activity in mouse kidneys was also significantly lower by 32.26% in the PDA@DG3@PEG@FA@DOX + PTT group (5.69 ± 2.20 µM/min/mg protein) compared to the PDA@DG3@PEG@FA group (8.40 ± 1.24 µM/min/mg protein) (Figure 8).

### 2.3. Anticancer Efficiency

**Figure 9:**
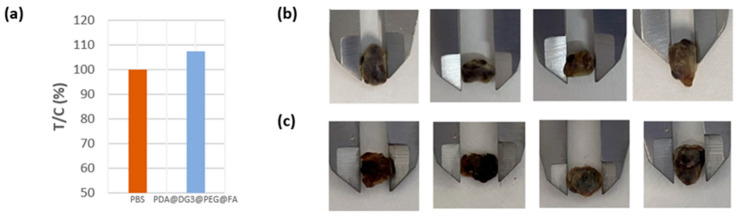
(**a**) Mean tumor volume in animals receiving PDA@DG3@PEG@FA over the control volume (T/C) 10 days after first injection; (**b**) pictures of measured tumors from animals receiving PDA@DG3@PEG@FA, and (**c**) pictures of measured tumors from animals receiving PBS.

The antitumor efficacy of the different treatments was assessed by tracking tumor growth; tumor sizes in each group were measured with calipers. To characterize antitumor activity, we used the following index: tumor volume versus control volume (T/C) [21]. PBS-treated mice showed tumor growth with an average tumor volume of about 180 cm^3^ 52 days after tumor induction, with the first tumors not being observed until 28 days after tumor cell inoculation. In the case of PDA@DG3@PEG@FA, it did not show antitumor activity; the results showed a 7.4% increase in tumor growth, with an average tumor volume of about 197 cm^3^ compared to PBS 10 days after the end of treatment (Figure 9a). The tumors in the treatment group PDA@DG3@PEG@FA@DOX + PTT were completely burned, leaving black scars at the original tumor sites. It has been reported that NIR light does not cause any damage to the tumor itself [9]. In our previous research, we also observed that magnetic materials with PDA for combined therapy, which are based on cellular NIR irradiation, do not cause any damage to cancer cells [22]. Tumor sizes in each group were measured with calipers. There was no difference in the tumors in the mice injected with PDA@DG3@PEG@FA compared to the tumors in the mice in the control group (Figure 9b,c).

## 3. Discussion

It is well known that size, shape, and modification of nanoparticles can influence a different toxicity effect in mice. However, every modification of given particles can cause a different toxicological effect. Their great potential in in vitro research requires testing on animals so that in the future it is possible to use their abilities in clinical trials. In this article, we have shown the change in oxidative stress marker levels in the organs of animals exposed to PDA nanoparticles with PAMAM dendrimers functionalized with folic acid in combined CT-PTT. We chose three important organs for our study, kidneys, liver, and brain, due to the large impact of oxidative stress on the activation of many intracellular signals inducing apoptosis or cell hypertrophy, and thus organ dysfunction. Nanomaterials based on PDA, due to the presence in various redox states and the ability to accept electrons, can generate free radicals, which affect the toxicity of PDA [18]. The resulting ROS can affect proteins, lipids, or nucleic acids, which leads to various side effects [23]. Oxidative stress acting on the body’s cells and products resulting from the reaction of molecules with free radicals contributes to the development of various diseases. For this reason, the parameters enabling the assessment of the biological redox state are important in the assessment of health and disease development [24]. According to some studies, cancer cells usually produce more ROS than normal cells. In normal cells, proliferation is dependent on ROS signaling. Cancer cells may be more susceptible to oxidative stress because they operate with elevated basal levels of ROS-mediated signaling, which is required for increased growth rates. Adding an agent that increases ROS production or decreases the ROS scavenging capacity can therefore push cancer cells past the breaking point in terms of lipid peroxidation, DNA damage, and protein oxidation [25]. Oxidative stress parameters such as TP, GSH, CAT, GST, TBARS, NO, and TEAC concentration allow us to assess the impact of free radicals on the body [24].

The ability of polydopamine to have an adsorption and covalent interaction with proteins was tested, and it was shown that they adsorbed on PDA films through various electrostatic, van der Waals, or hydrogen bonding interactions [26]. Covalent bioconjugation with proteins can occur when catechol groups are oxidized to quinones, which are susceptible to nucleophilic attack by amines and thiols via Michael addition and Schiff base reactions [27]. Li et al. studied mice administered intravenous polydopamine labeled with arginine-glycine-aspartic-cysteine acid (PDA-RGDC) with doxorubicin (PDA-RGDC-DOX) and phototherapy. The animals were administered PDA-RGDC-DOX at a concentration of 2 mg/mL and were exposed to an 808 nm laser at a dose of 2 W/cm^2^ for 5 min. They then tested five markers of liver function, including total protein. After a month, in the livers of mice, no significant differences in the concentration of total protein were found compared to the control group receiving PBS [28]. However, in the study of Zhang et al., which compared total protein concentrations, they focused on animals from the control group and animals receiving nanoparticles. To determine markers of liver function, total protein was chosen as one of the biochemical parameters tested in serum. As a result of the analysis, no statistically significant difference was observed between the group of Balb/c mice receiving PBS intravenously and the group of animals injected with nanoparticles synthesized from polydopamine and mesoporous calcium phosphate (PEG-ICG PDA/mCaP H-JNPs) at a dose of 20 mg/kg of to the tail vein. This may suggest that the administration of these nanoparticles did not result in obvious liver dysfunction [29]. In our study, the level of TP was compared in the liver, kidneys, and brain of the mice between the control group of animals, PBS, animals administrated with PDA@DG3@PEG@FA, and animals administrated with PDA@DG3@PEG@FA@DOX and exposed to an 808 nm laser at a dose of 1 W/cm^2^ for 5 min—PDA@DG3@PEG@FA@DOX + PTT.

Moreover, glutathione is widely distributed in the human body and plays a significant role in various physiological and pathological functions [30]. As an endogenous antioxidant, it can scavenge free radicals, maintain intracellular redox homeostasis, regulate the immune response, and participate in many key metabolic processes [31]. In addition, GSH is a marker reflecting human health, and abnormal levels of GSH in biological systems are strongly associated with many diseases, such as liver damage and cancer [32]. Cancer cells have a high level of oxidative stress, which makes them more sensitive to reduced glutathione deficiency, which can be used in cancer therapy [33]. In the studies of Feng et al., mice were injected with polydopamine into the tumor together with the photosensitizer, L-arginine, and methylene blue, which was supposed to target the nanomaterial to liver cancer. The study administered 50 µL of polydopamine at a dose of 1 mg/mL. There was a 21% reduction in GSH levels compared to the PBS control group [34].

Increased production of ROS in the body’s cells can cause, i.e., lipid peroxidation, which is quantified as the concentration of thiobarbituric acid reactive substances (TBARS). We observed that the concentration in the liver and kidney showed statistically significant differences in the lipid peroxidation of mice from the PDA@DG3@PEG@FA group in relation to two groups: control, PBS, and the PDA@DG3@PEG@FA@DOX + PTT group. TBARS concentration was also significantly higher in the PDA@DG3@PEG@FA@DOX + PTT group when compared to the group receiving PBS. Levels of TBARS were elevated in both treatment groups, as polydopamine nanoparticles and doxorubicin increase oxidative stress markers, but the group receiving both therapies had a significantly lower level of lipid peroxidation marker. In the study of Kuthati et al., the researchers gave 7-week-old Wistar rats mesoporous polydopamine as a morphine carrier to study its effect on free radical levels. A model of partial transection of the sciatic nerve was used to induce neuropathic pain. In the study, the MDA concentration representing lipid peroxidation in animals that had undergone partial transection of the sciatic nerve and received mesoporous polydopamine was 3.1 ± 0.34 nmol/mg protein, compared to 2.5 ± 0.51 nmol/mg protein in the control animals. These results were not statistically significantly different [35].

The measure of the total antioxidant capacity of Trolox equivalents (TEAC) determines the overall (enzymatic and non-enzymatic) antioxidant potential compared to the antioxidant capacity of Trolox, which is a synthetic analog of α-tocopherol with greater reducing capacity [36]. Our results showed that PDA@DG3@PEG@FA statistically decreased the level of TEAC compared to the control group, which seems to relate to polydopamine ABTS•+ scavenging properties. In a work on the analysis of hydrogels containing polydopamine in various concentrations, O’Connor et al. examined (in percent) the ability to reduce the cation radical ABTS•+ in relation to Trolox. As the concentration of PDA in the hydrogel increased, the percentage of reduced ABTS increased (O’Connor et al., 2020 [16]). Shi et al., in research on the synthesis and action of immunosuppressive nanoparticles in the treatment of ischemic stroke, used polydopamine as a carrier in the designed nanoparticle. They proved its concentration-dependent antioxidant properties by examining the ability to reduce ABTS•+ [37].

In addition, cancer cells often alter catalase expression, and it plays a dichotomous role in neoplastic processes. Catalase may protect cells from tumor initiation and progression by preventing the accumulation of ROS. A decrease in the expression and activity of the enzyme was observed, e.g., in lung and pancreatic cancer. On the other hand, other cancer cells have been shown to require high levels of antioxidant activity to compensate for high ROS production and prevent apoptosis. The associated increased catalase activity occurs, for example, in melanoma or glioblastoma [38]. From our research, it seems the level of catalase is significantly lower when animals also receive doxorubicin and photothermal therapy. Bai and Cederbaum, in their study, showed that catalase protects human HepG2 liver cancer cells from apoptosis caused by the administration of DNA-damaging cytostatics. Increased expression of catalase decreased the level of p53 protein, which is a suppressor protein involved in the apoptosis process and accelerated its degradation [39].

In the context of cancer therapy, these are preliminary studies to provide information about the potential effects of their therapies. During the course of therapy, it was not noticed that the tumor decreased enough to be considered an anticancer concept. Studies using polydopamine nanoparticles also did not cause tumor enlargement. Taking into account the oxidative stress studies, we can conclude that PDA-based nanoparticles may show weak toxic effects on the organs being tested. Therefore, the period during which the mice were tested may be important. Studies are needed that would allow us to observe animals for a longer period of time, also during the period of long-term toxicity.

Taken together, our results revealed that in the liver of animals, the TEAC parameter indicates an increased activity of antioxidant enzymes in the case of combined therapy compared to the control group. However, the TBARS parameter in the livers and kidneys indicates increased toxicity in the case of therapy with PDA@DG3@PEG@FA and PDA@DG3@PEG@FA@DOX + PTT compared to the control group. In the kidneys of animals, reduced levels of TEAC have been demonstrated in the group of animals exposed to PDA@GD3@PEG@FA compared to the group receiving PBS, so the reduced antioxidant capacity of Trolox equivalents may indicate reduced antioxidant protection. Some parameters, such as GST activity, CAT, or NO concentration in mice brains, indicate a reduced level of oxidative stress from the group that received functionalized nanomaterials compared to mice from the control group.

In our work, the most important was the effect of chemotherapy alone and combined chemo and phototherapy on the generation of stress in the organs through the method of treatment; however, this research has great potential and could be extended to include many more methods of treatment as well as a longer period of time or a larger research group. Our results provide a good representation of the in vivo toxicity of these polydopamine-based materials and may provide a starting point for further research on this material. Taking the obtained results into consideration, we are convinced that prepared PDA nanoparticles might serve as suitable multifunctional nanoplatforms for other cancer types, e.g., breast and ovarian, since their strong photothermal properties combined with their capacity of delivery of, for example, DOX, render them efficient nanoplatforms for oncological purposes.

## 4. Materials and Methods

### 4.1. Materials and Characterization

The particles were prepared according to the reported protocol [4], and their physical properties were in agreement with those in the article. The particles were characterized by transmission electron microscopy (TEM), and micrographs were recorded on a JEM-1400 microscope (JEOL, Tokyo, Japan) working at an accelerating voltage of 120 kV. Samples were drop cast on a copper grid (Formvar/Carbon, TedPella, Redding, CA, USA). Fourier transform infrared (FT-IR) spectra were recorded on a Vertex 70 spectrometer (Bruker, Mannheim, Germany) in KBr pellets. Zeta potentials and hydrodynamic diameters were measured using Zetasizer Nano ZS (Malvern Instruments Ltd., Malvern, UK). Photothermal and photostability experiments were performed at λ = 808 nm wavelength NIR laser at a power density of 2 W/cm^2^ (Changchun New Industries Optoelectronics Tech. Co., Ltd., Changchun, China).

### 4.2. Ethics Committee Approval

The study design was approved by the Ethics Committee for Animal Experiments Affairs in Poznań, Poland (Approval No. 20/2019 and 29/2020). Procedures concerning the handling and use of laboratory animals were performed in accordance with European Union (UE) regulations under Directive 2010/63/EU on the protection of animals used for scientific purposes. In order to protect the animals, the experiments were carried out in accordance with the so-called 3Rs principle (Replacement, Reduction, Refinement), conducting studies on the required minimum number of animals and maintaining the observation time so that the results remain consistent and statistically assessable. We collected all data in accordance with ARRIVE 2.0 guidelines. In vivo experiments were carried out in the Laboratory of Experimental Animals of the University Apparatus Center of the Poznań University of Medical Sciences, Poznań, Poland. The above-mentioned centers are entities listed by the Ministry of Education and Science. Contractors have individual permits to plan and conduct experiments, as well as to kill animals.

### 4.3. Animals and Experimental Treatments

The experiment was conducted with 18 female BALB/c nude mice with the following characteristics: CAnN.Cg-Foxn1nu/Crl, outbred herd, 5 weeks old, and an average body weight of 17.50 ± 1.30 g (Charles River Laboratories, Germany) For the purposes of the experiment, animals of one sex (female) were used in order to minimize the number of used animals and obtain reliable results. Animals were housed in polypropylene cages GM500 (Tecniplast, Buguggiate, Italy) (n = 2 mice/cage) with autoclaved aspen bedding 2 × 2 × 1 mm (Tapvei, Paekna, Estonia) under controlled environmental conditions (12 h light/12 h dark: 6 am–6 pm; temperature: 22 ± 2 °C; air humidity: 50–60%). In order to enrich the living conditions of the animals, shelters and blocks for grinding teeth (Tapvei, Estonia) were placed in the cages. The animals were acclimated to laboratory conditions two weeks before the start of the experiment (Experimental Animal Laboratory) with ad libitum access to water and nutritious feed. The animals were fed with the 1414 Forti formula (Altromin, Lage, Germany). During animal experiments, we respected all ethical issues related to working with animals in accordance with the guidelines of the Ethics Committee for Animal Experiments Affairs in Poznań, Poland. After two weeks of acclimation, HepG2 cells (2 × 10^6^ cells) in 100 μL Matrigel Matrix (Corning, Inc., Corning, NY, USA) were subcutaneously injected on the backs of all female BALB/c nude mice, CAnN.Cg-Foxn1nu/Crl. When the tumor volumes reached about 80 mm^3^, the animals were used in further research. We calculated tumor volume as (tumor length) × (tumor width)^2^/2.

### 4.4. In Vivo Treatments

Eighteen mice with tumors of about 80 mm^3^ were randomly divided into three groups (n = 6 per group), and they were injected one time into the tail vain with 100 μL of PBS, PDA nanoparticles coated with PAMAM dendrimers and functionalized with folic acid by PEG linker (PDA@DG3@PEG@FA), and PDA@DG3@PEG@FA with encapsulated doxorubicine (DOX) (PDA@DG3@PEG@FA@DOX), respectively. The injected doses were 35 mg/kg bw of PDA and 15 mg/kg bw of DOX. Then, 24 h after injection, the PDA@DG3@PEG@FA@DOX group also received laser treatment; the tumors of the mice treated with PDA@DG3@PEG@FA@DOX were irradiated once with NIR light (1.0 W/cm^2^) (Changchun New Industries Optoelectronics Tech. Co., Ltd., Changchun, China) for 5 min—PDA@DG3@PEG@FA@DOX + PTT (Figure 1). After treatments, the body weight and tumor sizes of each mouse were monitored by a caliper at a special time point every day for ten days. We calculated the relative tumor volumes as T/C (C was the tumor volume before treatments, and T represented the real-time measurement result of the tumor volume after treatments). To further explore the effect of therapy, 14 days after initiation of treatment, the animals were intraperitoneally administered a 1:1 (*v*/*v*) combination of ketamine (90 mg/kg; Kepro, Woerden, The Netherlands) and xylazine (10 mg/kg; Kela, Hoogstraten, Belgium) to induce anesthesia. Then, 10 min after injection, after they had achieved deep anesthesia, the animals were sacrificed by taking blood from the heart and removing the liver, kidney, and brain tissues. To measure the enzymatic activity of TP, GSH, GST, TBARS, TEAC, NO, and CAT, each rat tissue was placed in PBS and kept in a −80 °C freezer.

### 4.5. Preparation of Tissue Samples

Biochemical determinations were performed in the Laboratory of Environmental Research at the Department of Toxicology from the Poznan University of Medical Sciences. Amounts of 0.25 g of liver, kidney, and brain tissue were weighed. The biological material was placed in 50 mL Falcon tubes to which 2 mL of PBS buffer (Sigma-Aldrich, St. Louis, MO, USA) diluted 1:9 with saline was added. The tissues were comminuted in a homogenizer (24,000 rpm) and transferred to 15 mL Falcon tubes. The biological material was centrifuged for 10 min at 4 °C (4200 rpm). The resulting supernatant was transferred to 2 mL Eppendorf tubes and centrifuged again for 10 min at 4 °C (6000 rpm). The resulting supernatant was pipetted into 2 mL Eppendorf tubes. The biological material prepared in this way was stored in a freezer at −80 °C.

The methods described below have previously been used to assess oxidative stress on carbon nanotubes [40].

### 4.6. Determination of Total Protein (TP) Concentration

Total protein assays in the samples were performed by the method of Lowry et al. [41]. The test material was diluted with water that was redistilled 400 times. Two reactions were then carried out. The first was the reaction between the nitrogen atoms of the peptide bond and copper (II) ions in an alkaline medium. The samples were mixed and incubated for 10 min at room temperature. After the reduction of copper ions to Cu+ ions, Folin–Ciocalteu reagent (Sigma-Aldrich) was added. The samples were mixed and incubated for 60 min at room temperature, protected from light, resulting in the reduction of phosphotungstic acid and phosphomolybdic acid to the corresponding oxides. The absorbance was measured by the endpoint method at a wavelength of λ = 750 nm (Spectrophotometer UV-VIS 160, Shimadzu, Kyoto, Japan).

### 4.7. Determination of Reduced Glutathione (GSH) Concentration

GSH concentration was determined using the Ellman method [42]. An amount of 100 μL of the homogenates was mixed with 100 μL of a 4% sulfosalicylic acid solution (Sigma-Aldrich) and followed by the addition of 200 μL of Na_2_CO_3_ solution (Sigma-Aldrich). The samples were centrifuged for 10 min at room temperature at 3000 rpm. An amount of 800 μL of DTNB solution (Sigma-Aldrich) was added to 200 μL of the supernatant and mixed. After a 20 min incubation at room temperature, their absorbance was measured by the endpoint method at a wavelength of λ = 412 nm (Spectrophotometer UV-VIS 160, Shimadzu).

### 4.8. Determination of Lipid Peroxidation (TBARS) Concentration

Lipid peroxidation was measured using the method of Ohkawa et al. [43]. The reaction was carried out in an acidic environment and at an elevated temperature. To 100 μL of the homogenates, 500 μL of a solution of thiobarbituric acid (Sigma-Aldrich), trichloroacetic acid (Sigma-Aldrich), and hydrochloric acid (Sigma-Aldrich) was added. After incubation, the samples were cooled and 1000 μL of n-butanol was added followed by centrifugation for 8 min at 4000 rpm. The absorbance was measured by the endpoint method at a wavelength of λ = 532 nm (Spectrophotometer UV-VIS 160, Shimadzu).

### 4.9. Determination of Antioxidant Capacity Trolox Equivalents (TEAC)

TEAC was determined using the method of Re et al., which consists of measuring the ability of antioxidants in the test material to reduce the stable cation radical ABTS•+ in relation to the standard—Trolox [44]. ABTS (Sigma-Aldrich) and potassium persulfate (Sigma-Aldrich) were mixed in a ratio of 5:1. The mixture was incubated for 12 h at room temperature and protected from light. An amount of 10 μL of the test material and 1000 μL of the diluted mixture were mixed and incubated in the dark. The absorbance was measured by the endpoint method at a wavelength of λ = 734 nm (Spectrophotometer UV-VIS 160, Shimadzu).

### 4.10. Determination of Nitric Oxide (NO) Concentration

NO concentration was determined using the method modified by Kleinbongard et al. [45]. Zinc sulfate (Sigma-Aldrich) was added to 200 µL of the test material and centrifuged for 5 min at 4 °C—14,000 prm. EDTA (Sigma-Aldrich) and MES (Sigma-Aldrich) were then added to the supernatant, and after mixing, the sample was diazotized with sulfanilamide (Sigma-Aldrich) and combined with N-1-naphthylethylenediamine (Sigma-Aldrich) to produce a colored red-violet compound. The intensity of the color was measured spectrophotometrically at the maximum wavelength of λ = 540 nm (Spectrophotometer UV-VIS 160, Shimadzu).

### 4.11. Determination of S-Transferase Activity (GST) Concentration

Glutathione s-transferase activity was determined using the method of Habig et al. [46]. An amount of 200 μL of GSH (Sigma-Aldrich) was added to 1.7 mL of phosphate buffer (Sigma-Aldrich), and the samples were incubated for 5 min at 37 °C. After incubation in a heat block (Thermoblock TB-951U, JWElectronic, Warszawa, Poland), 100 μL of CDNB (Sigma-Aldrich) and 5 μL of test material were added. Absorbance was measured by the kinetic method in 60 s at a wavelength of λ = 340 nm (Spectrophotometer UV-VIS 160, Shimadzu).

### 4.12. Determination of Catalase (CAT) Concentration

Catalase activity is based on the decomposition reaction of hydrogen peroxide (H_2_O_2_) [21]. PBS was added to the H_2_O_2_ (Sigma-Aldrich) solution and stirred. The solution was incubated at 37 °C for 15 min (Thermoblock TB-951U, JWElectronic). An amount of 25 μL of the test material was pipetted into the quartz cuvette, and then the previously incubated solution was added to initiate the reaction. A decrease in absorbance was observed within 1 min at λ = 240 nm (Spectrophotometer UV-VIS 160, Shimadzu).

### 4.13. Statistical Analysis

All statistical tests were performed using Statistica 13 by TIBCO and PQStat by PQStat Software https://pqstat.pl/, Poznań, Poland. The significance level was α = 0.05. The normality of the distribution of variables was tested using the Shapiro–Wilk test. The Kruskal–Wallis test and the Conover–Iman multiple comparison test were used to compare parameters for variables not normally distributed. For variables normally distributed but not equal in variance, the Kruskal–Wallis test with the Conover–Iman multiple comparison test was used, and for variables normally distributed and equal in variance, the analysis of variance test for samples not related to the Fisher LSD test of multiple comparisons was used.

## Data Availability

Data are contained within the article.
